# Positive Association between Macular Pigment Optical Density and Glomerular Filtration Rate: A Cross-Sectional Study

**DOI:** 10.3390/jcm12165312

**Published:** 2023-08-15

**Authors:** Hiroki Tsujinaka, Keigo Saeki, Kenji Obayashi, Tomo Nishi, Tetsuo Ueda, Nahoko Ogata

**Affiliations:** 1 Department of Ophthalmology, Nara Medical University, Kashihara 634-8521, Nara, Japan; kinstar@naramed-u.ac.jp (H.T.);; 2 Department of Epidemiology, Nara Medical University, Kashihara 634-8521, Nara, Japan

**Keywords:** macular pigment optical density, estimated glomerular filtration rate, age-related macular degeneration

## Abstract

Although decreased macular pigment density is associated with the development of age-related macular degeneration (AMD), exactly how this decrease may contribute to the development of AMD is still not fully understood. In this study, we investigated the relationship between macular pigment optical density (MPOD) and estimated glomerular filtration rate (eGFR). MPOD was measured using MPS II (Electron Technology, Cambridge, UK) in 137 participants who showed no clinical signs of AMD at 3 months after cataract surgery, and simple and multiple linear regression analyses were performed to determine the associations with age, sex, abdominal circumference, diabetes, hypertension, smoking, intraocular lens color, visual acuity before and after surgery, and eGFR. The participants were divided into two groups based on the median MPOD (0.58): the high-pigment and low-pigment groups. The mean value of eGFR in the high-pigment group was significantly higher than that in the low-pigment group (64.2 vs. 58.1, *p* = 0.02). The simple linear regression analysis revealed a significant positive association between MPOD and eGFR (β = 0.0034, 95% confidence interval [CI]: 0.0011–0.0056, *p* = 0.0038), and this association was independent of age, sex, abdominal circumference, diabetes, smoking, hypertension, best-corrected visual acuity (BCVA) before surgery, BCVA after surgery, and intraocular lens color (β = 0.0033, 95% CI: 0.00090–0.0058, *p* = 0.0076). These results show a strong association of renal dysfunction with the decrease in MPOD.

## 1. Introduction

Macular pigments are composed of three types of xanthophylls: lutein, zeaxanthin, and meso-zeaxanthin [1]. Xanthophylls are nutrients that are present in the human body but cannot be synthesized in vivo [2]. Lutein, for example, is naturally found in yellow petals such as marigolds, green and yellow vegetables such as spinach, carrots, and pumpkin, as well as the green leaves of plants. Xanthophylls are absorbed in the small intestine and are mainly transported by binding to high-density lipoproteins. From the choroidal capillaries, they are incorporated into the photoreceptor outer segment through retinal pigment epithelial cells. Macular pigments are renowned for their antioxidant properties within the macula. They accumulate in the retina as a specific protein-binding protein and are thought to play a protective role because they scavenge the reactive oxygen species (ROS) generated in photoreceptor and pigment epithelial cells [3]. Furthermore, macular pigment absorbs short-wavelength visible light, including blue light, and thereby prevents the generation of reactive oxygen species [3]. Age-related macular degeneration (AMD) is an important cause of irreversible visual disorders, particularly in older adults. The number of AMD patients is estimated to be 196 million in 2020, projected to increase to 288 million worldwide by 2040 [4]. AMD is thought to be caused by age-related deterioration of the retinal pigment epithelium’s ability to process waste products, which results in the deposition of waste products in the macula, causing degeneration in the cells and tissues of the retina. The disease was traditionally classified into dry and wet types. The dry type is characterized by the presence of drusen, retinal pigment epithelial (RPE) cell degeneration, and progressive destruction of RPE cells. In contrast, the wet form is usually accompanied by greater visual loss and characterized by choroidal neovascularization led by several angiogenic cytokines, including vascular endothelial growth factor (VEGF). Wet AMD is not treatable, but owing to anti-vascular endothelial growth factor (VEGF), wet AMD has become a manageable disease. In addition, the involvement of Angiopoietin-2 (Ang-2), leading to vascular destabilization by inhibiting Angiopoietin-1 (Ang-1) and tyrosine kinase with immunoglobulin and epidermal growth factor homology do-mains 2 (Tie-2) signals, was actively reported in the pathogenesis of AMD, and bispecific antibodies against Ang-2 and VEGF have emerged as therapeutic agents that are attracting attention [5]. However, there are still many refractory cases, and further clarification of the pathogenesis and development of treatment is needed. Currently, there is no effective treatment for dry AMD, highlighting the importance of understanding the pathogenesis of dry AMD. Damage to the retina and choroid caused by ROS is thought to play an important role in the development of both dry and wet AMD [6]. Retina is known to be a tissue that consumes high oxygen to convert light into vision compared to other tissues, thus making it more susceptible to ROS compared to other tissues [7]. As a result, ROS, such as the superoxide (O_2_^•−^), hydroxyl radical (•OH), hydrogen peroxide (H_2_O_2_), and singlet oxygen (^1^O_2_) are more likely to accumulate around the retina [8]. Thus, controlling ROS could be a new strategy to prevent both dry and wet AMD. In the macula, the macular pigment is thought to play a central role in the control of ROS. In other words, macular pigment may help reduce production or lower levels of ROS. A decrease in macular pigment levels is thought to be involved in the development of AMD. A cross-sectional study on enucleated eyes reported that the amount of xanthophylls in the central 3 mm of the macula of eyes affected by AMD was approximately 63% of that in control eyes [9]. Obana et al. measured the level of macular pigment using resonance Raman spectroscopy as a cross-sectional study and showed that AMD patients had significantly lower macular pigment levels than control patients [10].

There are various methods for measuring macular pigment optical density (MPOD), which can be classified into two groups: psychophysical methods (which require a response from the subject) and objective methods (which require minimal input from the subject). In the psychophysical methods, there are two methods to measure the macular pigment level: heterochromatic flicker photometry (HFP) and minimum motion photometry. There are four objective methods, including fundus reflectometry, fundus autofluorescence, resonance Raman spectroscopy, and visually evoked potentials [11]. Of these, HFP is considered the standard method for macular pigment measurement [12], primarily because the measurement is simple, easy, and non-invasive to the patient. The HFP approach measures macular pigment levels based on changes in the flicker of light, and it is easily affected by the cataract condition. It was reported that higher carotenoid intake [13], younger age [14,15], male sex [16], high-iris-pigment volume [17], non-smoking history [18], and higher abdominal circumference [19] are associated with high-macular-pigment levels. However, since most previous studies were not able to exclude the influence of cataracts while measuring macular pigment levels, we aimed to measure the macular pigment levels of patients who had undergone cataract surgery 3 months ago and to explore the determinant of pigment level from the systemic condition.

Several kidney and eye diseases, including AMD, are reported to be interrelated because they share many structural and pathological characteristics [20]. Kier et al. reported that the renal glomerulus is anatomically similar to the outer retina, and several cytokines such as VEGF are implicated in the pathogenesis of both renal and retinal disease [21]. However, the relationship between renal dysfunction and retinal disease remains unclear. In this study, we investigated the relationship between pigment and renal function.

## 2. Materials and Methods

### 2.1. Study Design

This cross-sectional study was a sub-analysis of The Effect of Blue-Blocking Intraocular Lenses on Circadian Biological Rhythm: Protocol for a Randomized Controlled (CLOCK-IOL color) study. The study protocol was previously published [22] and registered in the University Hospital Medical Information Network Clinical Trials Registry (UMIN 000014559). This study was approved by the Ethics Committee of Nara Medical University (Approval Code: 13-032) and was designed in compliance with the Declaration of Helsinki. Informed consent was obtained from all participants.

### 2.2. Participants

Participants were patients aged 60 years or older who visited Nara Medical University and were diagnosed with grade 2 or higher-grade nuclear opacifications based on the Lens Opacities Classification System III [23]. The exclusion criteria were as follows: depression being treated with medication, severe mental illness or dementia, severe corneal opacity, severe glaucoma (visual field defect > −14 dB on the Humphrey Field Analyzer [Carl Zeiss AG, Oberkochen, Germany]), proliferative diabetic retinopathy, vitreous hemorrhage, macular edema, AMD, and combined cataract and glaucoma surgery or combined cataract surgery and vitrectomy. Of the 174 patients who gave consent to participate in the study, 37 patients were excluded because they did not perform all tests, including MPOD measurement and blood tests. One hundred and thirty-seven patients who fulfilled these criteria were finally included in the analysis.

### 2.3. Measurement of MPOD

In this study, MPOD levels were measured using MPS II, which uses the HFP approach. The measurement principle of MPS II was reported previously [24,25]. Briefly, this device recorded and analyzed the difference in sensitivity to flickering blue and green light. Blue light is absorbed by the macular pigment; therefore, if the amount of macular pigment is high, the participants will perceive greater flicker when blue light is projected alternately with the green light of the same intensity. To obtain MPOD, the blue output value (Lbc) and green output value (Lgc) of the equiluminance point at the fovea, and then the blue output value (Lbp) and green output value (Lgp) of the equiluminance point at the periphery were obtained, which theoretically had no macular pigment. The following formula was used to calculate the MPOD:

MPOD=klog10 (Lbc/Lgc)−klog10 (Lbp/Lgp)

In the MPS II, the peripheral area, located 8° nasally from the fovea, served as the reference area for zero macular pigment. MPS II detects macular pigment density in the outermost region of the indicator size used for measurement. The index size of MPS II is 1° on the retinal surface, allowing us to measure the amount of pigment at a distance of 0.5° from the fovea. MPS II has two inspection modes, “standard mode” and “detailed mode”. The standard mode measures only the fovea value without measuring the 8° periphery value. A predetermined value is used as the reference value for the periphery. This mode is easy to perform and takes a short time, making it suitable for screening. However, due to age-related decrease in lens permeability, the reference value in the periphery varies with age. Therefore, the obtained MPOD value is indicated as an “estimated value”. Standard mode cannot be used for spectacles with blue light cut lenses or for patients with yellow-colored intraocular lens (IOLs). Therefore, we used the detailed mode in our study. After measuring the MOPD value of the fovea, the peripheral 8° value was also measured. The measurement time was longer than the standard mode. The examination can be performed even with spectacles using blue light-cut lenses or with yellow-colored intraocular lenses. The obtained MPOD value was expressed as an absolute value. MPOD was measured in all patients 3 months after cataract surgery when the IOL was fixed in the lens capsule and visual acuity was stable. Measurement of MPOD was performed on the eye that was planned to undergo cataract surgery in a dark and quiet room.

### 2.4. Measurement of Estimated Glomerular Filtration Rate (eGFR)

Venous blood samples were collected from all participants, and serum creatinine concentrations were analyzed in Nara medical university hospital. eGFR was measured using two methods in accordance with previous reports [26]. First, eGFR was calculated according to the Japanese Society of Nephrology-Chronic Kidney Disease Practice Guide [27]. The following formula was used in men:

$$\begin{array}{l} eGFR\;(mL/min/1.73\;m^{2})\\ \;\;\;\;\;\;=194\times serum\;creatinine\;{\left[mg/dL\right]}^{-1.094}\times {\;age\;[years]}^{-0.287} \end{array}$$

The following formula was used in women:

$$\begin{array}{l} eGFR\;(mL/min/1.73\;m^{2})\\ \;\;\;\;\;\;=194\times serum\;creatinine\;{\left[mg/dL\right]}^{-1.094}\times {\;age\;[years]}^{-0.287}\\ \;\;\;\;\;\;\times \;0.739 \end{array}$$

The other method for eGFR calculation is the Chronic Kidney Disease Epidemiology Collaboration (CKD-EPI) formula for Japanese patients [28]. The following formula was used in men:

$$\begin{array}{l} eGFR\;(mL/min/1.73\;m^{2})\\ \;\;\;\;\;\;=0.813\times 141\times min\;(serum\;creatinine\;{\left[mg/dL]/\kappa ,\;1\right)}^{\alpha }\\ \;\;\;\;\;\;\times \;max\;(serum\;creatinine\;{\left[mg/dL]/\kappa ,\;1\right)}^{-1.209}\times {\;0.993}^{\;Age\;\left[years\right]}\\ \;\;\;\;\;\;\times \;1.018 \end{array}$$

The following formula was used in women:

$$\begin{array}{l} eGFR\;(mL/min/1.73\;m^{2})\\ \;\;\;\;\;\;=0.813\times 141\times min\;(serum\;creatinine\;{\left[mg/dL]/\kappa ,\;1\right)}^{\alpha }\\ \;\;\;\;\;\;\times \;max\;(serum\;creatinine\;{\left[mg/dL]/\kappa ,\;1\right)}^{-1.209}\times {\;0.993}^{\;Age\;\left[years\right]}\\ \;\;\;\;\;\;\times \;1.018 \end{array}$$

In the above-mentioned formulae, κ is 0.9 in men and 0.7 in women, α is −0.411 in men and −0.329 in women, min is the minimum serum creatinine level ([mg/dL]/κ or 1), and max is the maximum serum creatinine level ([mg/dL]/κ, 1 or 1).

### 2.5. Other Measurements

Slit lamp examinations were performed in all participants. Fundus photographs (TRC-50DX; Topcon Healthcare, Tokyo, Japan) and optical coherence tomography (Spectralis-OCT; Heidelberg Engineering, Heidelberg, Germany) images were obtained and analyzed by two independent ophthalmologists. In the case of disagreement between the two graders, the supervising grader was consulted to make the final diagnosis. The following information was acquired and investigated using self-reported questionnaires: age, sex, prevalence of diabetes mellitus, hypertension, and smoking history. We selected a yellow intraocular lens implant (IOL) (SN60WF or SN60AT; Alcon Inc., Geneva, Switzerland) and clear IOL (SA60AT; Alcon Inc.) in a 1:1 ratio for the cataract surgery through random assignment. All cataract surgeries were performed by the phacoemulsification and aspiration method using phaco instruments (Infinity or Centurion; Alcon Japan Ltd., Tokyo, Japan). The operation was performed with a small incision using an operating microscope (LUMERA 700; Carl Zeiss, Oberkochen, Germany) under sub-Tenon anesthesia. Intracapsular fixation of IOLs was performed by surgery. Postoperative examinations confirmed the absence of complications such as after cataract and postoperative inflammation. The best corrected visual acuity (BCVA) was measured by a skilled optometrist at 1 month before and 3 months after cataract surgery. The measured BCVA was converted to LogMAR values and used for the analysis. Abdominal circumference was measured one month before cataract surgery.

### 2.6. Statistical Analysis

Among the participants’ characteristics, continuous variables with normal distributions (age, sex, abdominal circumference, best-corrected visual acuity (BCVA), and eGFR) were expressed as mean and standard deviation (SD). All participants were classified into two groups (high- and low-pigment groups) based on the medial value of MPOD. Between the two groups, mean values and proportions were compared using t-test (for age, abdominal circumference, BCVA before surgery, BCVA after surgery, and eGFR) and Fisher’s exact test (for sex, diabetes, hypertension, current smoking and IOL color), respectively. We investigated the association between eGFR and the macular pigment level using simple and multiple linear regression models. To assess the independent association, we included age, sex, abdominal circumference, smoking history, hypertension, diabetes mellitus status, BCVA (LogMAR) before surgery, BCVA (LogMAR) after surgery, and IOL color as independent variables in multivariable models. We calculated the variance inflation factor (VIF) for all multivariable models and excluded multicollinearity with high VIF (>10). Statistical significance was set at *p* < 0.05. All analyses were performed using the GraphPad Prism 9 software (GraphPad Software, La Jolla, CA, USA).

## 3. Results

### 3.1. Baseline Characteristics

**Figure 1:**
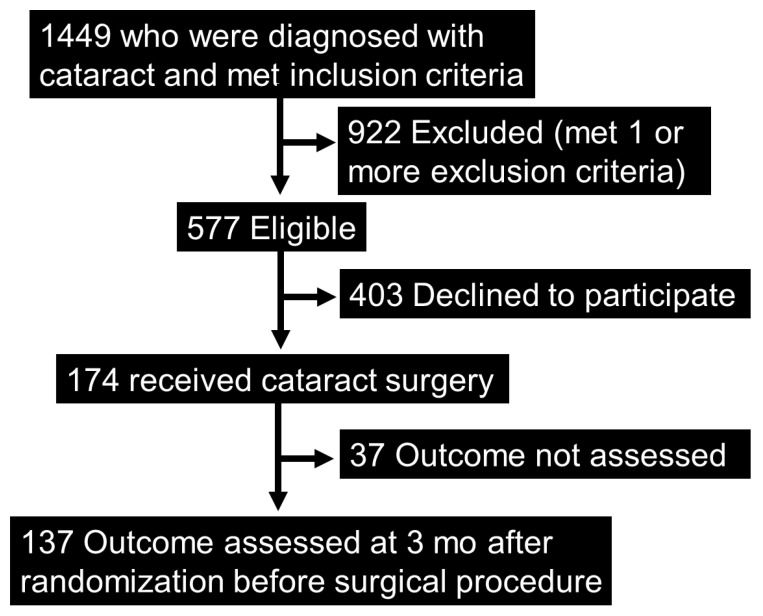
Study design flow diagram. A total of 137 participants were finally enrolled in our study.

A total of 2309 participants were newly diagnosed with cataracts at Nara Medical University Hospital from 1 July 2014 to 30 June 2017. Eligibility was examined in 2309 participants who met the inclusion criteria. Of the 1499 patients who matched the inclusion criteria, 922 were excluded according to the exclusion criteria. (Immediate cataract surgery, 512 participants; glaucoma, 101 participants; simultaneous glaucoma and vitreous surgery, 97 participants; major depression being treated with medication or severe mental illness or dementia, 87 participants; AMD, 73 participants; macular edema, 46 participants; proliferative diabetic retinopathy, 34 participants; severe corneal opacity, 32 participants; vitreous hemorrhage, 7 participants). Consequently, 577 participants met the study criteria, among whom 174 provided written informed consent. Since we were unable to obtain MPOD value 3 months after the surgery in the case of 32 participants and blood tests in the case of five participants, we conducted the final analysis on 137 participants. (Figure 1).

The mean age (SD) of the participants was 75 (6.6) years. Eighty-two participants were male (59.9%), and 55 were female (40.1%). The mean MPOD (SD) at 3 months after cataract surgery was 0.55 (0.22) optical density units (ODUs), and the mean eGFR of the participants was 61.2 (16.0) mL/min/1.73 m^2^ on using the Japanese Society of Nephrology-Chronic Kidney Disease Practice Guide formula and 62.3 (14.1) on using the CKD-EPI formula.

### 3.2. MPOD and eGFR

The participants were divided into two groups based on the median MPOD (0.58) at 3 months postoperatively: the high-pigment (N = 70) and low-pigment (N = 67) groups (Table 1). The eGFR in the high-pigment group was significantly higher than that in the low-pigment group on using both the Japanese Society of Nephrology-Chronic Kidney Disease Practice Guide formula (64.2 vs. 58.1, *p* = 0.02) and CKD-EPI formula (64.8 vs. 59.9, *p* = 0.04). 

**Figure 2:**
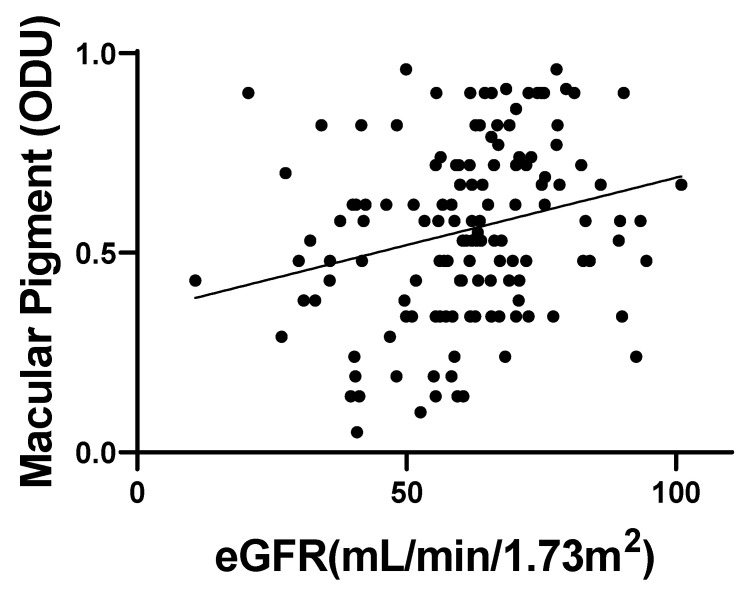
Simple linear analysis between MPOD and eGFR using the Japanese Society of Nephrology-Chronic Kidney Disease Practice Guide formula. (Regression Coefficient = 0.25, 95% CI: 0.081 to 0.40, *p* < 0.01). MPOD: macular pigment optical density, eGFR: estimated glomerular filtration rate.

The simple linear analysis revealed a significantly positive correlation between MPOD and eGFR when the Japanese Society of Nephrology-Chronic Kidney Disease Practice Guide formula was used (β = 0.0034, 95% CI: 0.0011–0.00056, *p* < 0.01) (Figure 2).

In multivariable analysis, the association between MPOD and eGFR stayed significant after adjusting for potential confounders (Model 1: adjusted for age and sex (β = 0.0034, 95% CI: 0.0011–0.00057, *p* < 0.01); Model 2: adjusted for age, sex, abdominal circumference, smoking history, hypertension, and diabetes mellitus status (β = 0.0035, 95% CI: 0.0011–0.00059, *p* < 0.01); Model 3: adjusted for age, sex, abdominal circumference, smoking history, hypertension, diabetes mellitus status, BCVA (LogMAR) before surgery, BCVA (LogMAR) after surgery, and IOL color (β = 0.0033, 95% CI: 0.0090–0.0058, *p* < 0.01). (Table 2).

## 4. Discussion

In this study, we found a positive relationship between MPOD and eGFR. Although several previous studies have reported the relationship between MPOD and general conditions [13,14,15,16,17,18,19], this is the first study to examine the association between macular pigment levels and renal function. The prevalence of eye disease and vision impairment is higher in individuals with chronic kidney disease (CKD) than those without CKD. For example, the incidence of diabetic retinopathy is known to be higher in patients with impaired renal function [20]. Furthermore, it was reported that patients with CKD are at a higher risk of developing AMD [29,30,31,32,33]. Anatomical similarities were noted, including the microvascular structure of the kidney, particularly as the microstructure at the interface of the capillary tuft, glomerular basement membrane, and the glomerular epithelial cells is similar to the structure of the eye, involving the choriocapillaris, Bruch’s membrane, and the retinal pigment epithelium. These similarities may explain why patients with renal impairment are more likely to have eye diseases [31]. Oxidative stress also may play a role in the development of AMD in the CKD patients. As CKD progresses, atherosclerosis is accelerated, making the body more susceptible to oxidative stress. These processes may explain the increased risk of AMD [31]. A negative correlation between AMD development and MPOD was previously reported [3]. The relationship between renal function and MPOD is unclear. 

This study has two strengths. First, we quantified MPOD correctly and examined its associated factors by including post-cataract surgery patients. Because MPS II, which was used to measure MPOD in this study, is a subjective test, the results are dependent on visual acuity. Most studies that have examined the correlation between systemic conditions and MPOD might not have correctly measured MPOD due to the presence of cataracts [13,15,16,17,18]. Several studies reported the correction methods for the measurement of MPOD values in the presence of cataracts, but such models have limitations [34,35,36,37]. Second, we calculated eGFR using two different methods. The eGFR formula is the simplified method. Although the CKD-EPI formula is the standard eGFR conversion formula used worldwide, it was reported that it overestimates the renal glomerular filtration rate when applied to the Japanese population [27]. Therefore, the Japanese Society of Nephrology-Chronic Kidney Disease Practice Guide formula, a calculation method developed by the Japanese Society of Nephrology and adapted to the Japanese physique, is commonly used in Japan. According to the Japanese Society of Nephrology-Chronic Kidney Disease Practice Guide, the accuracy of the eGFR formula recommended by them is such that 75% of cases fall within the range of ±30% of the measured GFR calculated using inulin and creatinine clearance [27]. For greater accuracy, this study examined the relationship between eGFR and MPOD values using two different eGFR formulas and found a significant correlation between eGFR and MPOD using either formula. These results strongly suggest that renal function correlates with macular pigment volume.

The mechanism by which macular pigment loss occurs remains unclear. However, one hypothesis on how MPOD is decreased in patients with low eGFR is the increase in blood reactive oxygen species (ROS). It is reported that oxidative stress caused by ROS is one of the causes of AMD [38], especially the absorption of excessive blue light by the retina producing large amounts of ROS [39]. Macular pigment may eliminate ROS induction by blue light [3], thereby preventing the development of AMD. MPOD may decrease with the continued presence of high levels of ROS in the body, such as from aging [15] and smoking [18]. CKD increases blood ROS levels [40], and the positive correlation between MPOD and eGFR observed in our study may have been mediated by elevated blood ROS. These results in our study may suggest that patients with impaired renal function benefit from active supplementation with xanthophylls, which increase macular pigment to prevent the development of AMD. This is because the macular pigment is considered to not be synthesized in the body [2]. In the Age-Related Eye Disease Study (AREDS), a prospective cohort study, lutein and zeaxanthin intake were determined using dietary questionnaires. The prevalence of exudative and dry AMD was significantly lower in the highest lutein and zeaxanthin intake group compared to the lowest lutein and zeaxanthin intake group [41]. In the AREDS2 study, supplementation with a combination of antioxidants, including lutein and zeaxanthin, was useful in preventing AMD [42]. AREDS-compliant supplements may be effective in preventing the development of AMD, especially in patients with impaired renal function, a high-risk group for AMD development, in two ways: by reducing ROS and replenishing macular pigment. Lutein and zeaxanthin are also taken up by the kidneys and act as antioxidants. Hu Y et al. reported [43] that high-level carotene dietary intake and serum concentration were associated with a lower mortality risk in the CKD population. AREDS-compliant supplements may also effectively protect kidney functions, resulting in a protective effect for macular pigment.

Our study had a few limitations. One limitation of this study was that lutein and zeaxanthin intake were not investigated in the self-reported questionnaires. Tanito et al. reported that 10 mg of lutein per day was effective in increasing MOPD levels by using resonance Raman spectrophotometry and one-wavelength autofluorescence imaging [44]. As mentioned before, since the macular pigment is entirely derived from diet, the intake of lutein and zeaxanthin may affect macular pigment levels. In this study, ROS accumulated in the body due to impaired renal function may have led to the decrease in macular pigment, but the actual ROS levels in the blood were not measured. Additional measurements are needed to investigate the relationships between MPOD values and systemic ROS concentrations. In addition, because the participants are all Asian in this study, the results may differ in other racial groups. Additionally, since our study was conducted in patients with post-cataract surgery, there may be an age bias. The average age of the patients in this study was 75, and even the youngest patient was 59. There were no young patients included in this study. Further research is needed to determine if the same is true for all generations. In our study, we only used one method, the HFP approach for measuring MPOD. This method is highly reproducible and provides data equivalent to fundus reflectometry approach [24]. However, recently, studies have shown that objective measurement of the macular pigment level using other methods are also highly accurate. Obana et al. reported that fundus autofluorescence spectroscopy approach using Spectralis OCT (Heidelberg Engineering, Heidelberg, Germany) was useful for measuring macular pigment level [14]. Since the target population in this study was predominantly elderly, a more accurate MPOD value could have been calculated by combining several measurement techniques, instead of using only subjective testing that might be influenced by cognitive function. As this is a cross-sectional study, the causal relationship between renal dysfunction and decreased MPOD is unknown. Longitudinal studies are required to substantiate our hypothesis.

## 5. Conclusions

In this study, we observed that MPOD and eGFR were positively correlated in patients with post-cataract surgery. One strength of this study was that MPOD values were measured in patients with IOL to rule out intermediate opacity. Additionally, we demonstrated a more accurate correlation between eGFR and MOPD by using two different eGFR conversion formulas. Macular pigment absorbs blue light, thereby reducing the generation of ROS from the retina. MPOD also removes ROS from the macula cells, and plays a role in preventing AMD. Supplementations that increase or maintains MPOD in patients, especially with impaired renal function, may help prevent AMD. Further investigation of the relationship between the patients’ systemic condition and MPOD is needed.

## Figures and Tables

**Table 1:** Baseline characteristics of 137 participants.

Characteristics	Pigment High	Pigment Low	*p*
Number (%)	70 (51.1)	67 (49.0)	
Age, mean (SD), years	74.9 (6.5)	75.2 (6.7)	0.82
Sex (men), number (%)	43 (61.4)	39 (58.2)	0.73
Abdominal circumference, mean (SD), cm	86.3 (9.5)	84.3 (9.6)	0.21
Diabetes, number (%)	10 (14.3)	13 (19.4)	0.50
Hypertension, number (%)	27 (38.6)	33 (49.2)	0.23
Current smoking, number (%)	27 (38.6)	25 (37.3)	1.00
IOL (Clear), number (%)	40 (57.1)	29 (43.2)	0.13
BCVA before surgery, mean (SD), logMAR units	0.48 (0.38)	0.48 (0.42)	0.97
BCVA after surgery, mean (SD), logMAR units	0.012 (0.11)	0.028 (0.13)	0.44
eGFR (SD), mL/min/1.73 m^2^	64.2 (15.3)	58.1 (16.3)	0.02
(CKD-EPI definition)	64.8 (12.8)	59.9 (15.1)	0.04

SD: standard deviation, IOL: intraocular lens, BCVA: best corrected visual acuity, MAR: minimum angle resolution, eGFR: estimated glomerular filtration rate, CKD-EPI: Chronic Kidney Disease Epidemiology Collaboration.

**Table 2:** Multiple linear regression analysis between MPOD and eGFR using three different models.

	β *	95% CI	*p*
Crude model	0.0034	0.0011–0.0056	<0.01
Adjusted model 1	0.0034	0.0011–0.0057	<0.01
Adjusted model 2	0.0035	0.0011–0.0059	<0.01
Adjusted model 3	0.0033	0.00090–0.0058	<0.01

Note: Model 1: adjusted for age and sex; Model 2: adjusted for age, sex, abdominal circumference, smoking history, hypertension, and diabetes mellitus status; Model 3: adjusted for age, sex, abdominal circumference, smoking history, hypertension, diabetes mellitus status, BCVA (LogMAR) before surgery, BCVA (LogMAR) after surgery, and IOL color. MPOD: macular pigment optical density, eGFR: estimated glomerular filtration rate, CI: confidence interval, IOL: intraocular lens, BCVA: best corrected visual acuity, MAR: minimum angle resolution, CKD-EPI: Chronic Kidney Disease Epidemiology Collaboration * β represents the regression coefficient, which indicates the change in MOPD value for a 1 increase in eGFR value.

## Data Availability

The data presented in this study are available upon request from the corresponding author. These data are not available to the public because they contain personal information of the participants.
